# Standardized measurement of dielectric materials’ intrinsic triboelectric charge density through the suppression of air breakdown

**DOI:** 10.1038/s41467-022-33766-z

**Published:** 2022-10-12

**Authors:** Di Liu, Linglin Zhou, Shengnan Cui, Yikui Gao, Shaoxin Li, Zhihao Zhao, Zhiying Yi, Haiyang Zou, Youjun Fan, Jie Wang, Zhong Lin Wang

**Affiliations:** 1 Beijing Institute of Nanoenergy and Nanosystems, Chinese Academy of Sciences, Beijing, 100083 P. R. China; 2 College of Nanoscience and Technology, University of Chinese Academy of Sciences, Beijing, 100049 P. R. China; 3 School of Materials Science and Engineering, Georgia Institute of Technology, Atlanta, GA 30332 USA; 4 School of Materials Science and Engineering, Tsinghua University, Beijing, 100084 P. R. China

**Keywords:** Devices for energy harvesting, Materials for devices, Electronic properties and materials, Electronic and spintronic devices, Characterization and analytical techniques

## Abstract

Triboelectric charge density and energy density are two crucial factors to assess the output capability of dielectric materials in a triboelectric nanogenerator (TENG). However, they are commonly limited by the breakdown effect, structural parameters, and environmental factors, failing to reflect the intrinsic triboelectric behavior of these materials. Moreover, a standardized strategy for quantifying their maximum values is needed. Here, by circumventing these limitations, we propose a standardized strategy employing a contact-separation TENG for assessing a dielectric material’s maximum triboelectric charge and energy densities based on both theoretical analyses and experimental results. We find that a material’s vacuum triboelectric charge density can be far higher than previously reported values, reaching a record-high of 1250 µC m^−2^ between polyvinyl chloride and copper. More importantly, the obtained values for a dielectric material through this method represent its intrinsic properties and correlates with its work function. This study provides a fundamental methodology for quantifying the triboelectric capability of dielectric materials and further highlights TENG’s promising applications for energy harvesting.

## Abstract (web summary)

Determining the triboelectric charge and energy density of dielectric materials is generally limited by many factors, failing to reflect their intrinsic behaviour. Here, a standardized strategy is proposed employing contact-separation TENG and supressing air-breakdown to assess max triboelectric charge and energy densities leading to an updated triboelectric series.

## Introduction

Considering the concerns about energy crisis, environmental pollution, and energy demand in the new era of internet of things and sensor networks, triboelectric nanogenerator (TENG) provides a superior solution to harvest wasted mechanical energy around our daily life for powering widely distributed electronics with a sustainable, clean and self-powered manner^1^. The high output and high energy conversion efficiency in low frequencies, combined with other advantages including wide materials choice, easy fabrication, low cost, light weight and flexibility, have successfully enabled TENG’s promising usage ranging from micro/nano power sources, self-powered sensors, flexible electronics to large-scale blue energy harvesting^2–5^. Triboelectric charge density (TECD) dominates the output capability of TENG^6^ and reflects the triboelectric performance of dielectric material. However, it relies on environmental factors such as atmosphere pressure^7–9^, temperature^10–12^, and humidity^13–15^. Moreover, theoretical results have demonstrated that TECD on dielectric’s surface is confined by breakdown effect^16,17^, and that’s why output performance of TENG can be enhanced by raising breakdown threshold in conventional methods including structural design^18,19^, environmental control^7,8,20^, and ultra-thin dielectric layers^17^. In this regard, the measured TECD in atmosphere condition is inaccurate, and undoubtedly difficult to reveal relationships between materials’ intrinsic properties and triboelectric behaviors. Although the faraday cup and kelvin probe force microscope can measure charge quantity with high resolution, charge loss can’t be fully eliminated, and the unavoidable sample transfer and limited choice of materials also heightened the testing difficulties. An attractive method is to quantify TECD of dielectric materials by liquid metal (solid–liquid triboelectric material pairs) in nitrogen^21,22^, but the breakdown effect can only be suppressed rather than fully removed in gas condition^9,23,24^, and the measured TECD is still an environmental-dependent quantity. More importantly, TECD of solid–liquid triboelectric material pairs can be only as a reference for solid–solid triboelectric material pairs, which can’t be completely equivalent^25,26^. Here the inaccurate TECD between solid–solid pairs still hampers the widespread promotion of TENG and the deep understanding of contact electrification.

On the other hand, as an energy harvester, performance improvement is always the most important and urgent research direction of TENG^27^. With the rapid advancement of TENG, developing universal and standardized methods to assess output capability of TENG still remain both highly desirable and a challenge. Although peak power density has been greatly elevated several orders to even megawatt per square meters in last decades and widely used for output capability evaluation^28^, it relies on device structure, working modes, and test factors such as velocity, acceleration, and frequency, which is not an ideal parameter to assess the output capability of TENG. Energy density, defined as the enclosed area of voltage-charge (*V*-*Q*) plot, has been demonstrated as one of the most standardized parameters that is intrinsically unaffected by test factors^6^. To maximize energy density, a promising direction is realizing open-circuit voltage firstly and then releasing charges in short-circuit, so the obtained energy density will approach to the maximum value^29,30^. However, air breakdown has been verified existing in any working modes of TENG^16,31^. Specifically, the maximum surface charge density in short-circuit is limited to 250 µC m^−2^ for the polytetrafluoroethylene (PTFE) film with the thickness of 50 µm^16^, while a lower threshold value in near open-circuit condition is only around 40–50 µC m^−2 ^^32^. Therefore, only a small portion of maximum energy density can be extracted in previous studies, so realizing maximum energy density is still a challenge. Moreover, to determine the existence of air breakdown in atmosphere condition, a reference value of TECD and energy density is critical and necessary, which shows profound guidance on improving performance of TENG. Therefore, an effective standardized strategy that can assess the maximum TECD and energy density of different materials could be an important step towards designing high-performance TENG, and shed light on the comprehensive understanding of contact electrification relating to materials intrinsic properties.

Here, a universal standardized strategy is proposed to quantify maximum TECD and energy density of dielectric materials in TENG. We systematically studied the effects of environmental factors and material parameters including atmosphere pressure, temperature, and dielectric’s thickness on the maximum surface charge density. The results not only reveal the great effect of air breakdown on charge loss in atmosphere condition, but also demonstrate the independence of maximum surface charge density and dielectric’s thickness in vacuum condition, paving the foundation to quantify TECD of various materials. Based on contact-separation TENG (CS-TENG) and vacuum environment, TECD of more than forty dielectric materials are assessed, and a record-high TECD of 1250 µC m^−2^ is realized between polyvinyl chloride (PVC) and copper. Furthermore, the elastic fluororubber was chosen to intimately contact different metals, and the results suggest that vacuum TECD may be related to the work function of contact metals. It is worth noting that vacuum TECD has three advantages: (1) as a reference for determining the presence of air breakdown in atmosphere condition; (2) revealing the relationship between TECD and material’s intrinsic properties; (3) utilizing to quantify the maximum energy density of dielectric materials. Then, without surface charges loss even in open-circuit condition, fifteen materials’ maximum energy density are assessed. This work provides a universal standardized strategy for assessing the maximum TECD and energy density of dielectric materials in TENG, which is helpful to bridge the gap between real output performance and maximized output performance, and shows potential to promote the understanding of contact electrification.

## Results

### Strategies for assessing triboelectric performance of dielectric materials

**Fig. 1:**
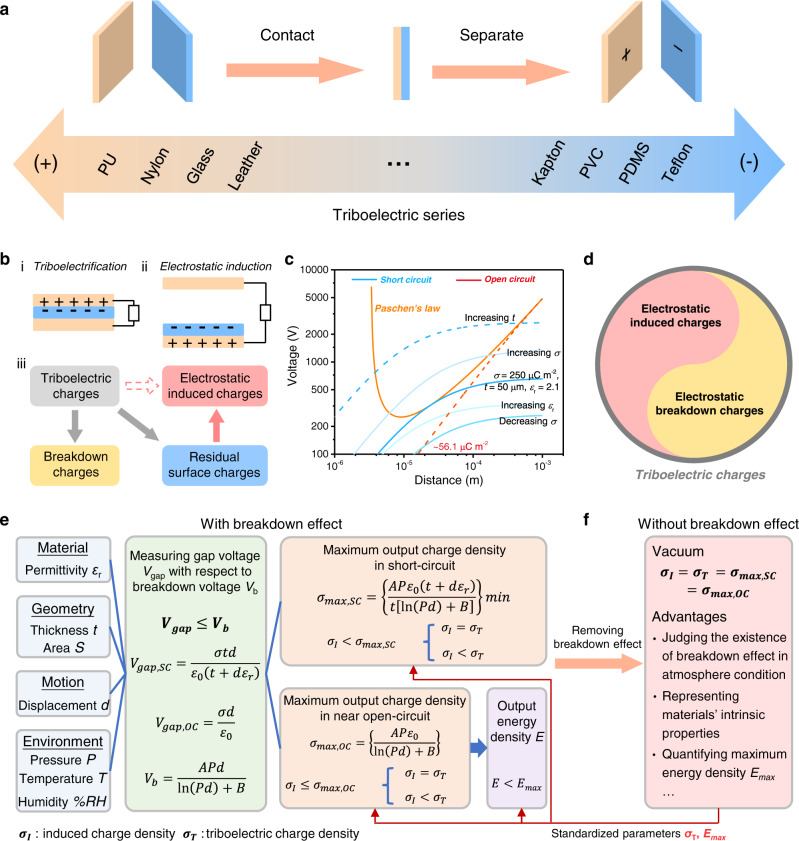
Strategies for assessing triboelectric performance of dielectric materials. **a** Represented qualitative triboelectric series. PU, polyurethane; PVC, polyvinyl chloride; PDMS, polydimethylsiloxane. **b** Schematic diagram shows working mechanism and charge transferring process of TENG. **c** Relationship of breakdown voltage and gap voltage across the triboelectric layers at various distances in TENG. **d** Schematic diagram shows the relationship between triboelectric charges and induced charges with the existence of breakdown effect. **e** The comprehensive strategy for assessing TECD with the existence of breakdown effect. Especially in open-circuit, it is difficult to restrict breakdown effect. **f** Taking the vacuum as an example showing the strategy for assessing TECD after removing breakdown effect, and the potential advantages of TECD in vacuum. Source data are provided as a Source data file.

Triboelectric series is a common tool to evaluate triboelectric property of different materials, and the material on the ends of the series will be more prone to lose or accept electrons, which indicates that the triboelectric series is qualitative because only the polarity of materials is considered (Fig. 1a and Supplementary Note 1)^33^. For the promising energy harvester-TENG, besides the charge transferring direction, the charge quantity is more important for designing high-performance TENG. Initially, we usually supposed that the electrostatic induced charges in external circuit is equal to the charges created by triboelectrification (Fig. 1b). However, as the deep understanding of working mechanism of TENG, it has been demonstrated that air breakdown widely exists in any working mode of TENG. More importantly, triboelectric charges will be released by air breakdown before they can be completely induced in external circuit, so the induced charges generally are smaller than real triboelectric charges. It is worth noting that triboelectric charges may also cause the ionization of atmosphere and attract opposite charges in atmosphere with trace amount and slow speed rather than spark discharge, which part of charge loss can hardly be detected.

Therefore, a comprehensive understanding of breakdown effect on output performance of TENG is needed. As shown in Fig. 1c, we plot the general breakdown curve described by Paschen’s law and gap voltage between two triboelectric layers in TENG with the relationship of separating distance. By changing the parameters relating to the gap voltage, the breakdown point (the intersection of gap voltage curve and breakdown curve) will be moved, so the output performance of TENG can be improved by regulating these parameters in previous studies such as decreasing dielectric’s thickness^17^, increasing relative permittivity^34^, increasing gas pressure^8^, and choosing the gas with high breakdown threshold^8^, etc. Especially in open-circuit condition, theoretical results indicate that maximum surface charge density is restricted to a very low value^32^. Overall, TECD are very prone to be affected by the above-mentioned factors, which results in measured induced charges smaller than real triboelectric charges (Fig. 1d). Those factors can be mainly classified into four parts: material parameter including permittivity *ε*_r_; geometry parameters including thickness *t* and area *S*; motion parameter including displacement *d*; and environment parameters including atmosphere pressure *P*, temperature *T* and humidity %*RH* (Fig. 1e).

In atmosphere condition with the possibility of air breakdown, all the above parameters should be carefully considered as much as possible, and the air gap voltage *V*_gap_ must be smaller than the breakdown voltage *V*_b_ during the whole working distance of TENG. Taking the negatively charged dielectric layer as an example, triboelectric charges transferring from the metal to the dielectric layer occurs at contact process based on contact electrification, whereas induced charges transferring in external circuit occurs at separating or contacting process based on electrostatic induction (Supplementary Fig. 1 and Supplementary Note 2). The physical model of breakdown effect in CS-TENG clearly indicates that air breakdown will cause surface charge loss and then decrease the induced charge density *σ*_I_ (Supplementary Fig. 2 and Supplementary Note 3). Generally, the short-circuit maximized theoretical charge density *σ*_max,SC_ can be derived as shown below (Supplementary Fig. 3 and Supplementary Note 4)^16,17^:

1 $${\sigma }_{{\max },{{{{\rm{SC}}}}}}=\left\{\frac{{AP}{\varepsilon }_{0}\left(t+{\varepsilon }_{r}d\right)}{\left[{{{\rm{ln}}}}\left({Pd}\right)+B\right]t}\right\}{{\min }}$$

where *A* and *B* are gas constants, and *P*, *ε*_0_, *d*, *ε*_r_, *t* are atmosphere pressure, vacuum permittivity (*ε*_0_ ~ 8.85 × 10^−12 ^F m^−1^), gap distance, relative permittivity and thickness of the dielectric layer, respectively. In near open-circuit condition, electrons in top electrode can’t timely transfer to the bottom electrode in external circuit, and the voltage across the air gap is independent of the dielectric’s thickness (Supplementary Fig. 4), so the maximized theoretical charge density in near open-circuit (*σ*_max,OC_) is limited by the following equation:

2 $${\sigma }_{{\max },{{{{\rm{OC}}}}}}=\left\{\frac{{AP}{\varepsilon }_{0}}{\left[{{{\rm{ln}}}}\left({Pd}\right)+B\right]}\right\}{{\min }}$$

For a general working distance range of CS-TENG, *d* is 0.01 m, and *σ*_max,OC_ is limited to only around 50 µC m^−2^ by air breakdown (Supplementary Note 4)^32^.

In this context, the induced charges are discussed from two aspects (Fig. 1e). In short-circuit condition, *σ*_I_ is less than *σ*_max,SC_, so *σ*_I_ will equal to TECD (the symbol is denoted as *σ*_T_) or less than *σ*_T_, where the condition of equal sign is that *σ*_T_ does not decay in this environment. In near open-circuit condition, for the triboelectric material with very weak triboelectric performance, *σ*_I_ is smaller than *σ*_max,OC_, and *σ*_I_ will equal to *σ*_T_; for the triboelectric material with strong triboelectric performance (*σ*_T_ > *σ*_max,OC_), *σ*_I_ is still limited to *σ*_max,OC_ by air breakdown, so *σ*_I_ will be smaller than *σ*_T_. Therefore, the strategy for assessing the maximum TECD with the existence of breakdown effect should carefully analyze these complex factors, especially for the condition where measured *σ*_I_ closes to *σ*_max,SC_. Despite that, it is still very difficult to obtain the true *σ*_T_ only by *σ*_I_ in atmosphere condition, because we still need to know in advance the true *σ*_T_ for judging whether there is charge loss. More importantly, for assessing maximum energy density, CS-TENG should be separated in open-circuit where the maximum surface charge density is limited to only around 50 µC m^−2^, so the maximum energy density *E*_max_ is still unknown and cannot be achieved with the existence of breakdown effect^6,30^.

It is clearly that *σ*_I_ will equal *σ*_T_, if breakdown effect is removed (Supplementary Note 5). Taking the vacuum environment as an example, the maximum surface charge density will also keep stable whether in short-circuit or open-circuit (i.e., *σ*_I_ =*σ*_T _= *σ*_max,SC_ = *σ*_max,OC_) because air breakdown is completely avoided (Fig. 1f). Besides, environmental factors and adsorbed water molecules on the delectric surface can also be removed in high vacuum as much as possible, avoiding charge loss from environmental factors. Therefore, obtaining *σ*_T_ in vacuum environment has several advantages for TENG, including: (1) judging whether there is breakdown effect in atmosphere condition; (2) revealing the relationship between *σ*_T_ and materials’ intrinsic properties; (3) using for quantifying materials *E*_max_, which will be discussed in detail in the following sections.

### Demonstration the feasibility of the proposed strategy

**Fig. 2:**
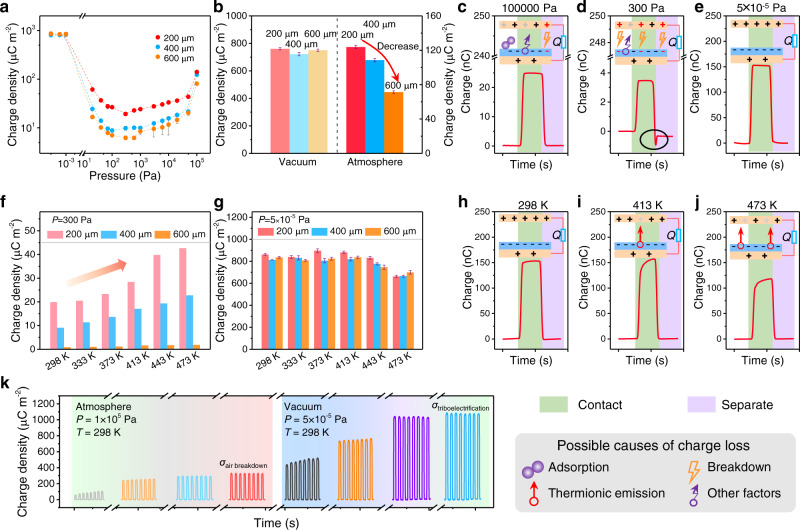
Demonstration the feasibility of the proposed strategy. **a** Surface charge density of the CS-TENG at various atmosphere pressures when the thickness of the PTFE film is 200 µm, 400 µm, and 600 µm. **b** Surface charge density of the CS-TENG in vacuum and atmosphere condition when the thickness of the PTFE film is 200 µm, 400 µm, and 600 µm. These results indicate that the surface charge density of CS-TENG is strongly related with the dielectric thickness in atmosphere condition, while it has no relationship with the dielectric thickness in vacuum condition. Represented curves of output charges of CS-TENG in **c** 100,000 Pa, **d** 300 Pa, and **e** 5 × 10^−5 ^Pa as well as corresponding charge dissipation model. **f** Surface charge density of the CS-TENG with different temperatures in the pressure of 300 Pa when the thickness of the PTFE film is 200 µm, 400 µm, and 600 µm. **g** Surface charge density of the CS-TENG at various temperatures in high vacuum when the thickness of the PTFE film is 200 µm, 400 µm, and 600 µm. Represented curves of output charges of CS-TENG at **h** 298 K, **i** 413 K, and **j** 473 K as well as corresponding charge dissipation model. **k** Steady surface charge density of PVC limited by air breakdown in atmosphere condition and triboelectrification in vacuum condition at 298 K. Error bars represent standard deviation, *n* = 5 independent replicates. Source data are provided as a Source data file.

A home-made high vacuum system was built to provide a reliable test environment (Supplementary Fig. 5), and the CS-TENG technique is used as a tool to reveal effects of three represented parameters on *σ*_I_: atmosphere pressure, dielectric’s thickness, and temperature. Taking the commonly used PTFE dielectric layer as an example, we can plot the relationship of *σ*_max,SC_, *P,* and *t* at different *d* (0–0.01 m, the general working range of CS-TENG) (Supplementary Fig. 6). Figure 2a and Supplementary Fig. 7 are the corresponding experimental results, which are in accordance with the theoretical results. Analyses based on the above results lead to three major conclusions: (i) both *P* and *t* have great effect on *σ*_I_ with the existence of air breakdown; (ii) the thinner the dielectric layer, the higher *σ*_I_ in atmospheric pressure and low vacuum conditions, which agrees well with the reported values^17^ (Supplementary Note 6); (iii) *σ*_I_ is independent with *t* in high vacuum condition (here, ~5 × 10^−5 ^Pa) (Fig. 2b), which makes it possible to quantify various materials with different thicknesses. To understand the three statements comprehensively, the represented single output charge waveform at atmosphere pressure (100,000 Pa), relative low pressure (300 Pa), and high vacuum (5 × 10^−5 ^Pa) conditions are plotted in Fig. 2c–e, respectively, where inset figures depict the corresponding charge dissipation model. At atmosphere pressure, the charge loss is caused by multiple factors including adsorbing gas molecules, air breakdown, and other factors (humidity, dust, etc.), while at 300 Pa, the charge loss is dominated by the breakdown effect. As shown in Supplementary Fig. 6b, the critical gap distance where breakdown occurs varies with *P*, showing a competing relationship between electrostatic induction and electrostatic breakdown. As pressure decreases to around 300 Pa, the possible breakdown point increases and the special breakdown signal can be detected during the separating process (the black circle in Fig. 2d, Supplementary Fig. 8, and Supplementary Note 7). Until high vacuum condition, *σ*_I_ keeps stable and is independent of dielectric thickness because air breakdown has been completely avoided.

Furthermore, considering complex effects of temperature on charge loss with the form of enhancing electrostatic breakdown and thermionic emission simultaneously, we compared *σ*_I_ at various temperature in atmosphere (with air breakdown) and vacuum condition (without air breakdown), respectively. To clearly illustrate it, *σ*_I_ of CS-TENG with different PTFE thicknesses was systematically studied at 300 Pa where air breakdown is most likely to occur. As shown in Fig. 2f, with temperature increase, *σ*_I_ of different PTFE thickness all increases because elevating temperature will move the breakdown point toward left and raise the critical breakdown voltage to some extent (Supplementary Fig. 9 and Supplementary Note 8). As shown in Fig. 2g, whereas in high vacuum condition (around 5 × 10^−5 ^Pa), *σ*_I_ almost remains stable with the increasing of temperature up to 400 K (Fig. 2h, i), and then decreases with temperature further enhanced to 473 K (Fig. 2j). The corresponding output voltage shows the same tendency, as depicted in Supplementary Fig. 10. These results indicate that thermionic emission may decrease the measured *σ*_I_, if the temperature is high enough (Supplementary Fig. 11 and Supplementary Note 9). When temperature is below around 400 K, thermionic emission is not obvious, so *σ*_I_ keeps nearly stable as shown in Fig. 2h (Supplementary Note 10). Therefore, we can conclude that *σ*_I_ is very close to *σ*_T_ with breakdown effect and thermionic emission avoided as much as possible (here, *P*: 5 × 10^−5 ^Pa and *T*: 298 K). The represented curve that *σ*_I_ gradually stabilize with time increasing is a strong evidence to demonstrate this conclusion (Fig. 2k). In addition, we also provide the charge decay curve of the representative PTFE film in vacuum condition (Supplementary Fig. 12 and Supplementary Note 11). An important deduction is that *σ*_I_ is often restricted by air breakdown in atmosphere condition, while *σ*_I_ in vacuum can be used to represent *σ*_T_, providing a reference to judge whether there is breakdown effect in atmosphere condition.

### TECD of various materials in vacuum condition

**Fig. 3:**
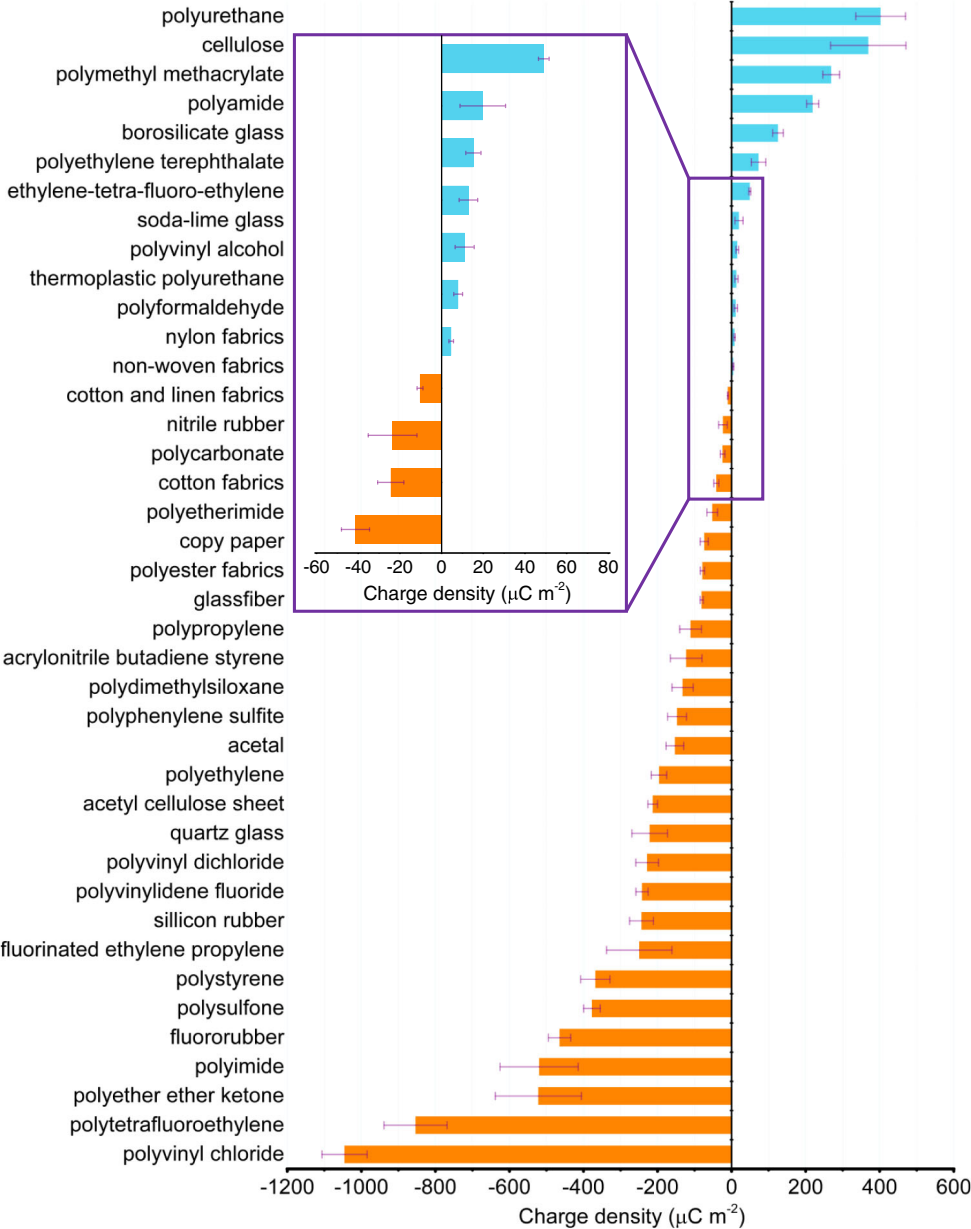
TECD of various materials in vacuum condition. Error bars represent standard deviation, *n* = 5 independent samples. Source data are provided as a Source data file.

By avoiding charge loss from air breakdown and thermionic emission effects, the standardized strategy was used to test more than forty dielectric materials’ TECD with the counterpart of Fe electrode, because the Fe electrode shows high-temperature stability and high hardness which can reduce materials transfer as much as possible (Supplementary Fig. 13 and Supplementary Notes 12–13). The initial surface charges on triboelectric layers are removed by absolute ethyl alcohol. We also test five materials with different thicknesses, and further demonstrate that *σ*_T_ indeed has no relationship with dielectric thickness here (Supplementary Fig. 14 and Supplementary Note 14). Moreover, to eliminate occasionality of testing a single sample, every kind of material was tested with five different samples, and the corresponding values were recorded and plotted in Fig. 3 (Supplementary Table 1). The negative and positive charge density indicates that this material will be negatively or positively charged, when it contacts with Fe electrode. We can find that polyvinyl chloride (PVC) and PTFE tend to obtain electrons while polyurethane (PU) and cellulose tend to lose electrons.

Different from the conventional triboelectric series only considering the polarity of materials, both of polarity and amounts of transferred charges are considered in our work. Moreover, by removing the effects of breakdown effect and environmental factors, here the tested *σ*_I_ can be considered as *σ*_T_, which can be used as a reference to judge the breakdown effect in atmosphere condition. More importantly, the quantified triboelectric series can better reflect materials intrinsic properties, and it is possible to reveal some relationship between *σ*_T_ and materials intrinsic properties.

### Effect of counterpart metal on triboelectric properties of dielectric materials

**Fig. 4:**
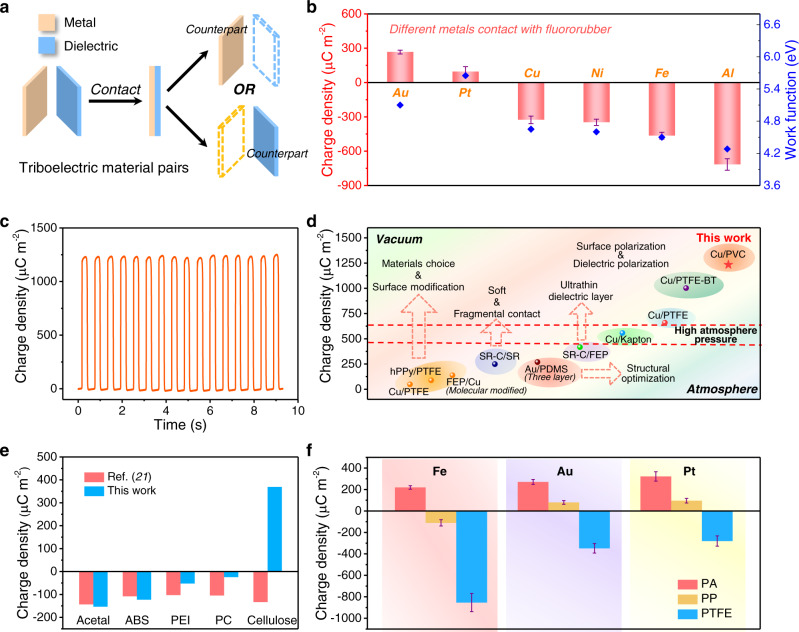
Effect of counterpart metal on triboelectric properties of dielectric materials. **a** Triboelectric material pairs with a metal or a dielectric as the counterpart. **b** TECD of fluororubber with the counterpart of different metals and their corresponding work functions. **c** TECD of PVC and Cu. **d** Summary of TECD records and corresponding realizing methods of the represented works and this work. The corresponding adopted materials is depicted in the circle. **e** Comparison of the TECD between the previous reported work and this work using the same five dielectric materials (Acetal, ABS, PEI, PC, and cellulose). **f** TECD of three dielectric materials (PA, PP, and PTFE) with the counterpart of three metals (Fe, Au, and Pt). Error bars represent standard deviation, *n* = 5 independent samples. Source data are provided as a Source data file.

Previously, many works have focused on studying triboelectric material pairs of a metal with different dielectric materials (Fig. 4a), while little attention was focused on a dielectric layer contact with different metals, because it is commonly assumed that the type of metals only has little impact on triboelectric performance in atmosphere condition. Having established the reliable method for quantifying dielectric material’s triboelectric property, we try to investigate the relationship between counterpart metal and *σ*_T_. To realize conformal contact between the dielectric material and counterpart metals, the elastic fluororubber was chosen as the counterpart due to its high elasticity and chemical stability. As shown in Fig. 4b, when fluororubber contacts with metals with different work functions, *σ*_T_ varies from around 266 µC m^−2^ changing to −736 µC m^−2^. The results also suggest that *σ*_T_ in vacuum has a certain relationship with the work function of the metals. In addition, *σ*_T_ of triboelectric material pairs of PVC-Cu can be up to around 1250 µC m^−2^ (Fig. 4c), obviously higher than the *σ*_T_ of triboelectric material pairs of PVC-Fe. Previously, extensive efforts have been made to improve charge density from materials choice^21^, surface modification^35^, soft and fragmental contact^18^, structural optimization^19^, ultra-thin dielectric layer^17^, high vacuum, and high atmosphere pressure environment^7,8^, and the highest *σ*_T_ was raised to 1003 µC m^−2^ between Cu-PTFE/BT (BT: Barium Titanate) (Fig. 4d and Supplementary Note 15). Although the effective charge density can be further enhanced to 3.53 mC m^−2^ in charge pump technology^20^ and to 8.8 mC m^−2^ in direct-current TENG (DC-TENG) with 50 DC units^36^, the real TECD in these TENGs is still relatively low and we still don’t know where is the upper limit of triboelectrification. By properly choosing triboelectric material pairs of PVC-Cu, we have raised this ceiling again and demonstrate the possibility to realize a higher *σ*_T_, showing a greater potential of triboelectrification (Supplementary Note 16).

In addition, we also compared our results with the previous reported works using the same five dielectric materials (Acetal; ABS: acrylonitrile butadiene styrene; PEI: polyetherimide; PC: polycarbonate; and cellulose.)^21^ to show the effect of breakdown effect on charge loss and the difference between solid–liquid pairs and solid–solid pairs. As shown in Fig. 4e, it is obvious that *σ*_T_ between our work and the previous work are very different, especially for the cellulose material, which shows the value of −133 µC m^−2^ in previous work (cellulose contact with Hg) but 369.06 µC m^−2^ (cellulose contact with Fe) in this work. The Acetal and ABS have higher TECD while the PEI and PC have lower TECD in this work, which are also very different from the results in previous work, verifying the *σ*_T_ of solid–liquid pairs and solid–solid pairs are not completely equivalent. The most represented triboelectric material of PTFE even shows an order of magnitude difference of *σ*_T_ between the previous work (−113.06 µC m^−2^)^21^ and our work (−853.7 µC m^−2^), reminding us to consider air breakdown effect when tests TECD in gas condition again.

Based on the results in Fig. 3 and Fig. 4b, we carefully chose three metals (Fe, Au, and Pt) with distinct work function differences to contact with three dielectric materials (PA: polyamide; PP: polypropylene; and PTFE) and forming nine triboelectric material pairs. The results in Fig. 4f show some regularity. PP is charged negatively against Fe, positively against Au, and more positively against Pt, so the effective work function of PP is probably between the work function value of Fe and Au. For PTFE, it is charged negatively against Pt, little more negatively against Au, and much more negatively against Fe, so the effective work function of PTFE seems higher than the work function of Pt, which agrees with the reported value of 5.8 eV^37^. PA is charged positively against Fe, little more positively against Au, and the most positively against Pt, so the effective work function of PA seems lower than the work function of Fe. The corresponding schematic diagram and detailed TECD values are shown in Supplementary Fig. 15. In addition, there are also some inconsistent reports, and the possible reasons are the influence of environmental factors and complex surface conditions. These results suggest the relevance of *σ*_T_ to the work function of counterpart metal again, which also reflect that the dielectric material could have the effective work function like the work function of metal. This provides the possibility to find relationships between TECD and material’s intrinsic properties in future studies.

### Energy density measurement of dielectric materials based on vacuum TECD

**Fig. 5:**
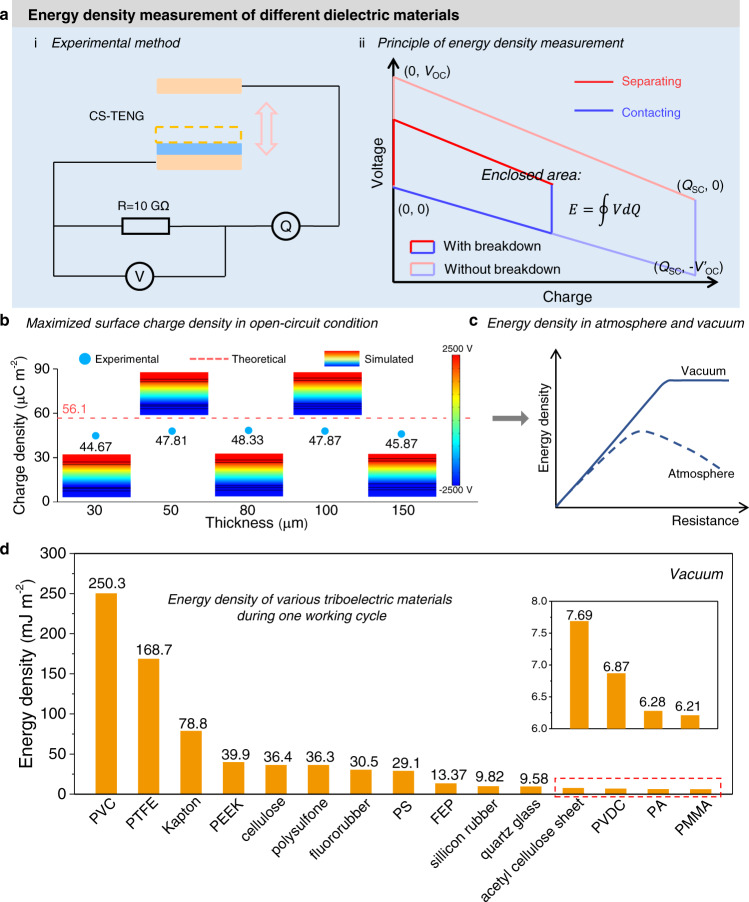
Energy density measurement of dielectric materials based on TECD in vacuum. **a** Energy density measurement method and principle. i Experimental method; ii principle. **b** Theoretical, simulated and experimental results of maximum surface charge density in open-circuit condition. **c** Schematic diagram shows the maximum energy density in atmosphere and vacuum condition. **d** Quantified maximum energy density of fifteen represented materials during one working cycle in CS-TENG. Source data are provided as a Source data file.

As the most important standardized figure-of-merit to quantify the performance of TENG, the maximum energy density of dielectric material still cannot be measurement so far^30^. The predominate reason is the unavoidable breakdown effect of TENG in atmosphere condition, especially for the CS-TENG. Energy density of TENG is described by *V*-*Q* plot, and the enclosed area of *V*-*Q* plot represents the output energy density. To realize the maximum energy density, the CS-TENG should be separated in open-circuit condition and then the charges transferring in external circuit. Comparing with the optimum matching resistance of general CS-TENG around tens to hundreds megohm, a resistance of 10 GΩ is connected in series in the test circuit, which is very equivalent to the open-circuit condition (Fig. 5ai). The measuring principle of maximum energy density with and without air breakdown effect is shown in Fig. 5aii. It is clearly that the commonly measured maximum energy density is much smaller than the real maximum energy density, because air breakdown effect restricts the surface charge density to a very low value when the CS-TENG works in open-circuit condition. With the existence of air breakdown, the theoretical, simulated, and experimental results of maximum surface charge density in Fig. 5b all indicate that the maximum surface charge density in open-circuit is only around 50 µC m^−2^ regardless of the thickness of dielectric layer, and the conventional methods such as materials choice, structural optimization, and ultra-thin dielectric layer are useless to restrict air breakdown here. Therefore, due to the output charges varying with the external load resistance in atmosphere^38^, the final output energy density will be obviously different in atmosphere and vacuum condition (Fig. 5c and Supplementary Note 17). In vacuum environment, based on the premise of *σ*_I_ = *σ*_T_ = *σ*_max,SC_ = *σ*_max,OC_, the output energy density is closer to the maximized value, providing a potential standardized strategy for quantifying maximum energy density of dielectric materials in TENG. As shown in Fig. 5d, the energy density of fifteen represented materials in CS-TENG during one working cycle is quantified (Supplementary Fig. 16), which is clearly much higher than the reported value and closer to the theoretical values, exhibiting the great potential of TENG for energy harvesting. For example, the current highest energy density of 110 mJ m^−2^ during one working cycle can be realized with an efficient power management strategy in previous work^39^, while the maximum energy density of 250.3 mJ m^−2^ can be achieved after avoiding air breakdown. In future, we need to find the method to restrict breakdown effect in CS-TENG (especially the breakdown effect in open-circuit) at atmosphere condition and simultaneously decrease the effect of parasitic capacitance on open-circuit voltage^40,41^, which will further enhance the output energy density of CS-TENG to a higher stage.

## Discussion

In summary, we propose a universal and standardized strategy to quantify the maximum TECD and energy density of dielectric materials in TENG. In atmosphere condition with the existence of air breakdown, we need to carefully analyze the effects of various parameters on air breakdown including materials parameter: permittivity; geometry parameters: thickness and area; motion parameter: displacement; and environment parameters: atmosphere pressure, temperature, and humidity, etc., so as to quantifying material’s triboelectric performance accurately as much as possible. Even then, we still not know the true TECD and maximum energy density as references. By circumvent these limitations in vacuum environment and combining with CS-TENG, the maximum TECD and energy density of dielectric materials in TENG can be quantified, and the vacuum TECD could be as a reference for determining the presence of breakdown in atmosphere condition. Then, we assessed the TECD of more than forty materials, and a high TECD of 1250 µC m^−2^ is realized between triboelectric material pairs of PVC-Cu, elevating the ceiling of TECD to a new record. With the elastic fluororubber as counterpart to contact different metals, the results suggest that TECD may be related to the work function of contact metal, revealing the relationship between TECD and the material’s intrinsic property. Moreover, without surface charges loss even in open-circuit condition, fifteen materials’ maximum energy density are assessed, exhibiting the great potential of TENG for energy harvesting. This research not only proposes a universal standardized strategy for assessing the maximum TECD and energy density of dielectric materials in TENG, but also provides the methodology for bridging the gap between real output performance and maximized output performance, which is helpful for designing high-performance TENG and the relevant researches relating to contact electrification.

## Methods

### Fabrication of the high-temperature resistant CS-TENG

Part I: A round shape of PTFE sheet was cut as the substrate (diameter: 20 mm; thickness: 3 mm) using a laser cutter (PLS6.75, Universal Laser System). A Fe plate (diameter: 20 mm; thickness: 0.1 mm) was adhered on the surface of the substrate as the electrode layer. Part II: Cut a round shape of PTFE sheet as the substrate (diameter: 15 mm; thickness: 3 mm). A Fe plate (diameter: 15 mm; thickness: 0.1 mm) was adhered on the surface of the substrate as the electrode layer, and the PTFE film was adhered on the surface of the electrode layer as the triboelectric layer. The Fe electrode was obtained by tailoring the Fe plate and treated by the sandpaper. Both metal plates were connected by high-temperature resistant wires for electrical measurement.

### Fabrication of the CS-TENG with different triboelectric materials

Part I: A square shape of acrylic sheet was cut as the substrate (length of side: 30 mm; thickness: 3 mm) using a laser cutter (PLS6.75, Universal Laser System). A Fe plate (length of side: 30 mm; thickness: 0.1 mm) was adhered on the surface of the substrate as the electrode layer. Part II: Cut a round shape of acrylic sheet as the substrate (diameter: 11.3 mm; thickness: 3 mm). A Fe plate (diameter: 11.3 mm; thickness: 0.1 mm) was adhered on the surface of the substrate as the electrode layer, and the triboelectric material was adhered on the surface of the electrode layer as the triboelectric layer with the area of 1 cm^2^. For the CS-TENG with different triboelectric pairs, the fabrication process is the same as the above method.

### Characterization

The experimental system was based on a home-made high vacuum system with a heater for building a stable working environment. A mechanical pump was used for realizing a low atmosphere pressure, and an adjustable air intake was combined to achieve pressure regulation. A molecule pump was used for realizing a high vacuum condition (the ultimate vacuum of the molecular pump is around 5 × 10^−5 ^Pa). Before test, the samples were placed in the high vacuum system until to the ultimate vacuum of the molecular pump, and keeping 2 h for steady state with the vacuum gauge closed (because the vacuum gauge can produce little ions affecting the environmental condition). When temperature changes, there will be 2 h for realizing equilibrium state. The vacuum was monitored by a low vacuum ionization gauge and a high vacuum ionization gauge. The temperature was monitored by a vacuum thermocouple gauge. The output charges and voltage of the CS-TENG are measured by an electrostatic electrometer (Keithley 6514). NI-6218 was used for data collection and the LabVIEW was used for realizing real-time data acquisition analysis. The energy density was calculated by the ratio of output energy to the geometric area.

## Supplementary information

Supplementary Information

## Data Availability

The authors declare that all the data that support the findings of this study are available within the article and its supplementary information files. Source data are provided with this paper.

## Source data

Source Data
