# Supplementary material for: Standardized measurement of dielectric materials’ intrinsic triboelectric charge density through the suppression of air breakdown
Source: Nat Commun. 2022 Oct 12;13:6019. doi: 10.1038/s41467-022-33766-z (PMC9556570; doi:10.1038/s41467-022-33766-z)
Supplement: Supplementary file 1 — Supplementary Information [file 41467_2022_33766_MOESM1_ESM.pdf]

Supplementary information for

Standardized measurement of dielectric materials' intrinsic triboelectric charge density through the suppression of air breakdown

Di Liu[1,2,δ], Linglin Zhou[1,2,δ], Shengnan Cui[1,2,δ], Yikui Gao[1], Shaoxin Li[1,2], Zhihao Zhao[1,2], Zhiying Yi[1], Haiyang Zou[3], Youjun Fan[4], Jie Wang[1,2]*, Zhong Lin Wang[1,2,3]*

[1]Beijing Institute of Nanoenergy and Nanosystems, Chinese Academy of Sciences, Beijing 100083, P. R. China.
[2]College of Nanoscience and Technology, University of Chinese Academy of Sciences, Beijing 100049, P. R. China.
[3]School of Materials Science and Engineering, Georgia Institute of Technology, Atlanta, GA 30332, USA.
[4]School of Materials Science and Engineering, Tsinghua University, Beijing 100084, P. R. China.

[δ]D. Liu, L. Zhou and S. Cui contributed equally to this work.
*Corresponding Author: J. Wang: wangjie@binn.cas.cn;
Z. L. Wang: zhong.wang@mse.gatech.edu

This file includes:
Supplementary Figure 1-16
Supplementary Note 1-17
Supplementary Table 1
References

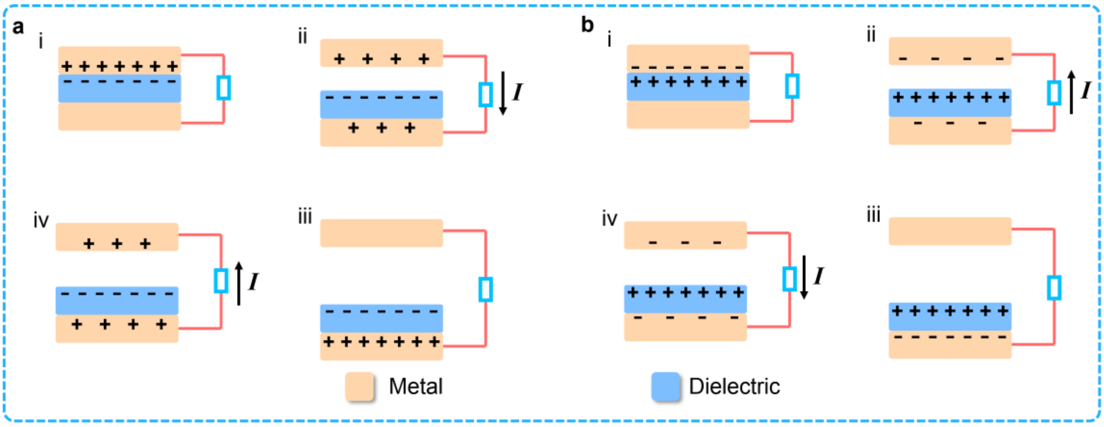

Supplementary Figure 1. Working mechanism of the CS-TENG. a The negatively charged dielectric layer. **b** The positively charged dielectric layer.

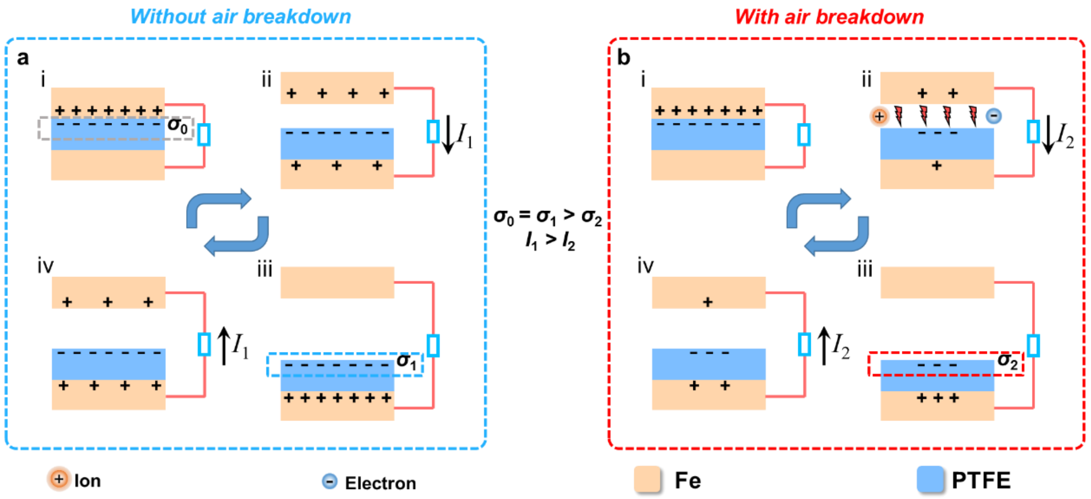

Supplementary Figure 2. The detailed working mechanism of CS-TENG with and without air breakdown effect. a Without air breakdown. **b** With air breakdown.

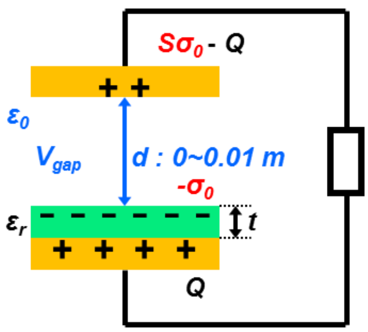

Supplementary Figure 3. Physical parameters of the CS-TENG in short-circuit.

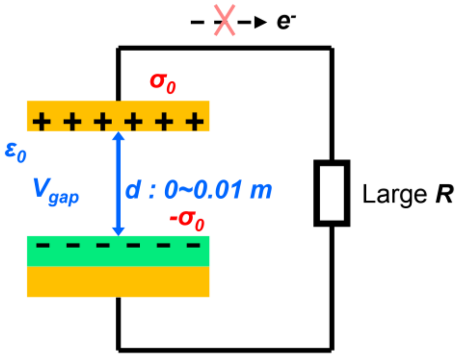

Supplementary Figure 4. Schematic of gap voltage of CS-TENG in near open-circuit condition.

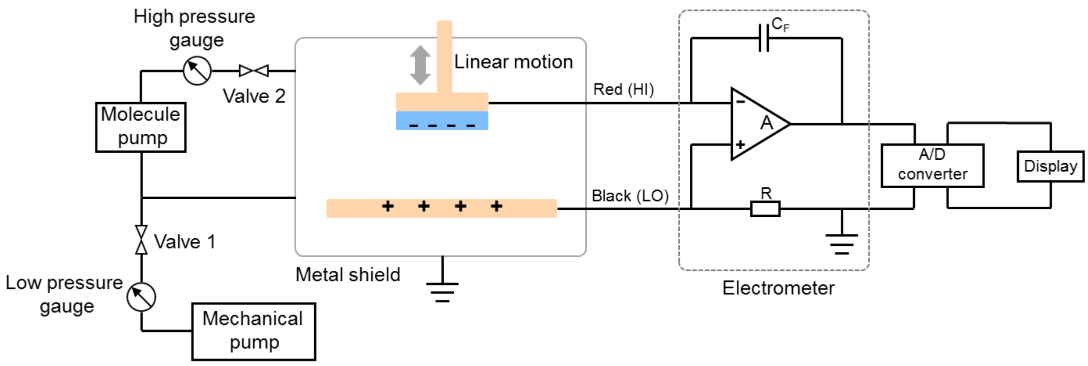

Supplementary Figure 5. The charge density measuring equipment and method.

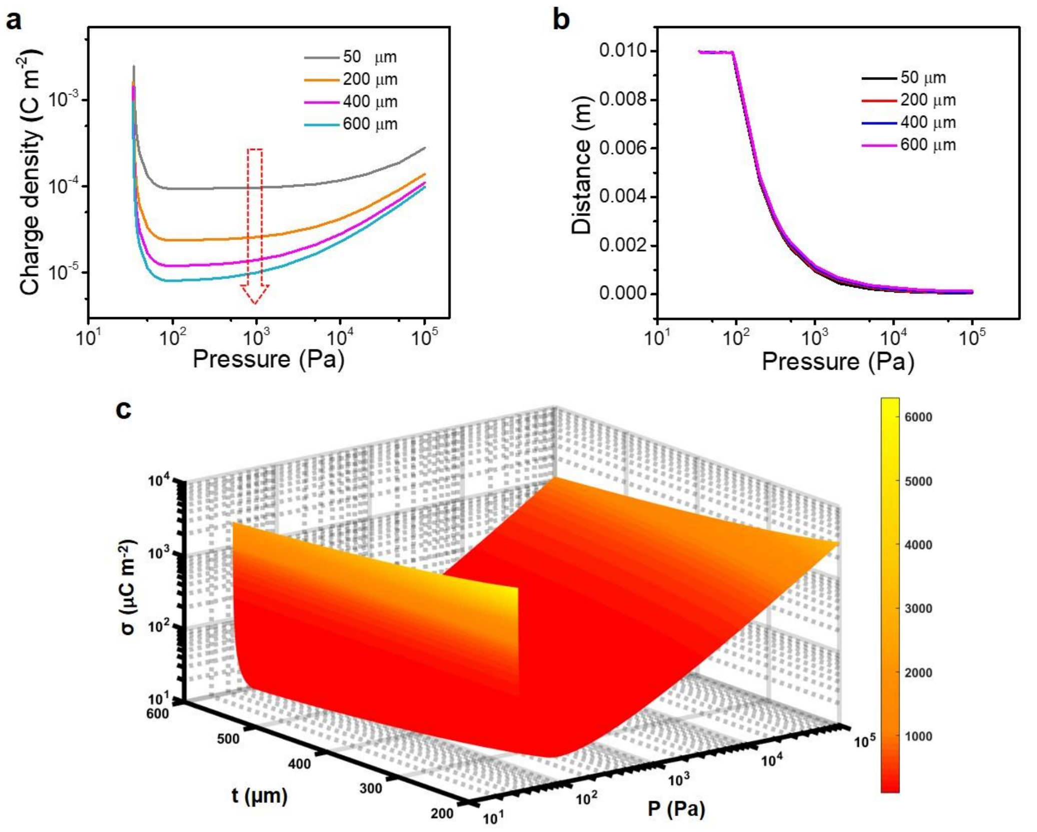

Supplementary Figure 6. The relationship of maximum surface charge density, atmosphere pressure and the thickness of the dielectric layer. a Maximum surface charge density at various atmosphere pressures. **b** The corresponding critical gap distance which the air breakdown is about to occur at various atmosphere pressures. **c** The relationship of maximum surface charge density, atmosphere pressure and the thickness of the dielectric layer. Source data are provided as a Source Data file.

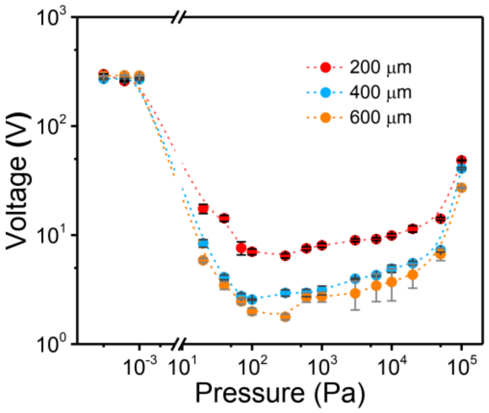

Supplementary Figure 7. Output voltage of the CS-TENG at various atmosphere pressures when the thickness of the PTFE film is 200 μm, 400 μm and 600 μm. Error bars represent standard deviation, $n = 5$ independent replicates. Source data are provided as a Source Data file.

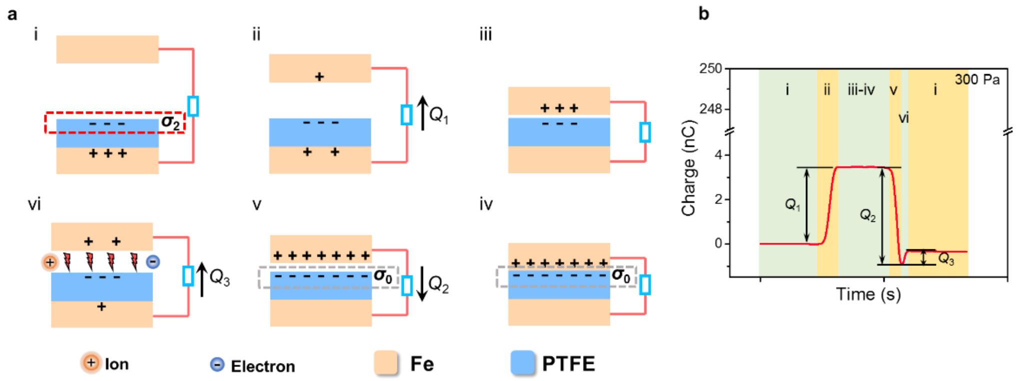

Supplementary Figure 8. The charge transfer process of the CS-TENG with breakdown effect happened. a The working mechanism of CS-TENG at around 300 Pa. **b** Output charge of CS-TENG at around 300 Pa. Source data are provided as a Source Data file.

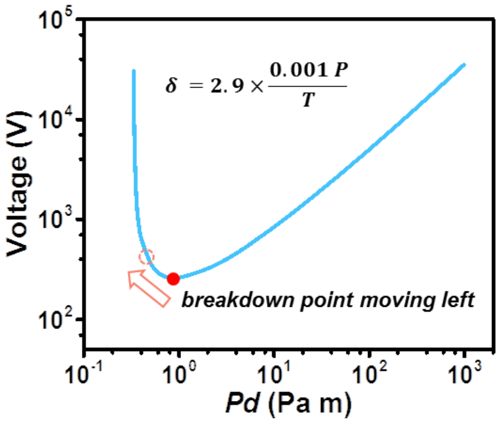

Supplementary Figure 9. Breakdown point of CS-TENG at 300 Pa moving left when temperature increases. δ represents the relative air density. According to the breakdown theory, the effects of changing temperature on breakdown voltage is reflected as the relative changes in air density (δ), which is defined as the ratio of the real density to the density under standard atmosphere condition. Given that the atmosphere pressure of 300 Pa is the critical point, temperature increase will reduce δ, and the breakdown voltage will increase. Source data are provided as a Source Data file.

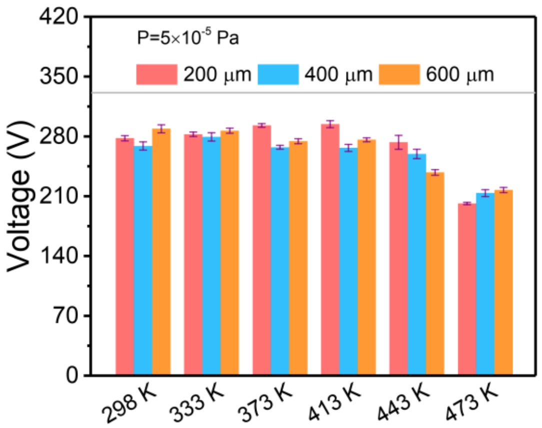

Supplementary Figure 10. Output voltage of the CS-TENG at various temperatures in high vacuum when the thickness of the PTFE film is 200 μm, 400 μm and 600 μm. Error bars represent standard deviation, $n = 5$ independent replicates. Source data are provided as a Source Data file.

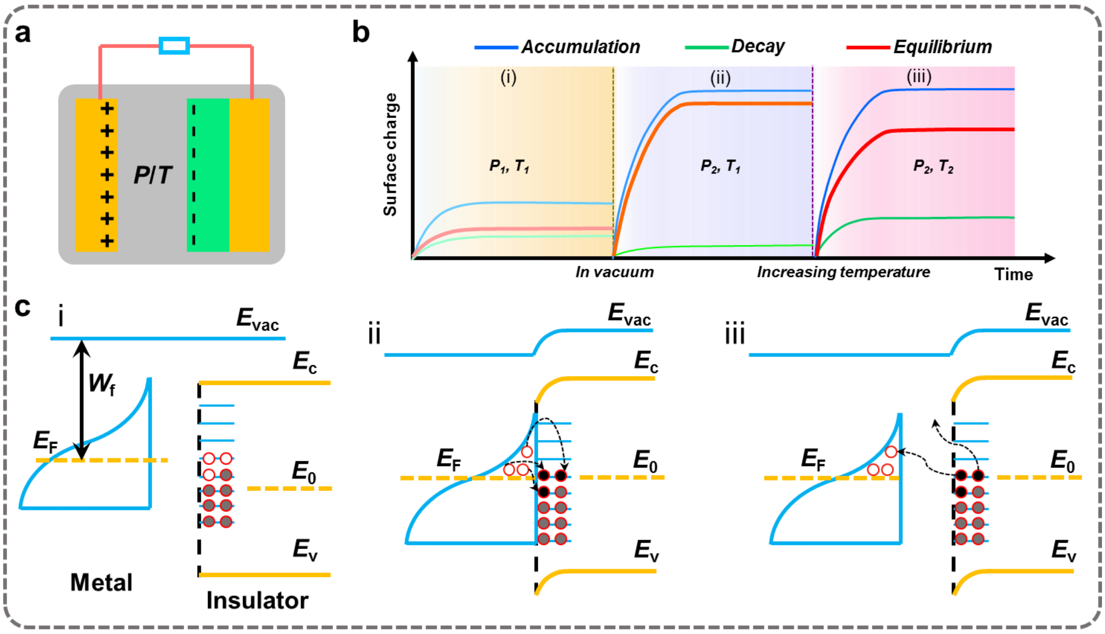

Supplementary Figure 11. The model for explaining effects of atmosphere pressure and temperature on the output performance of CS-TENGs. a Illustration the measuring electric circuit with the changeable atmosphere pressure and temperature. **b** The schematic diagram shows the distribution of surface charges at various atmosphere pressures and temperatures. P_1: the atmosphere pressure with the existence of air breakdown; P_2: the atmosphere pressure with the air breakdown avoided. T_1: low temperature with the thermionic emission not obvious; T_2: high temperature with the thermionic emission obvious. **c** Surface state models for explaining CE at high temperature for a case that E_F is higher than E_0. (E_{vac}, vacuum level; E_F, Fermi level; W_f, work function; E_C, conductive band; E_V, valance band; E_0, the highest occupied surface state level of the dielectric layer.) The charge transfer before contact (i), in contact (ii) and after contact (iii) between a metal and a dielectric layer.

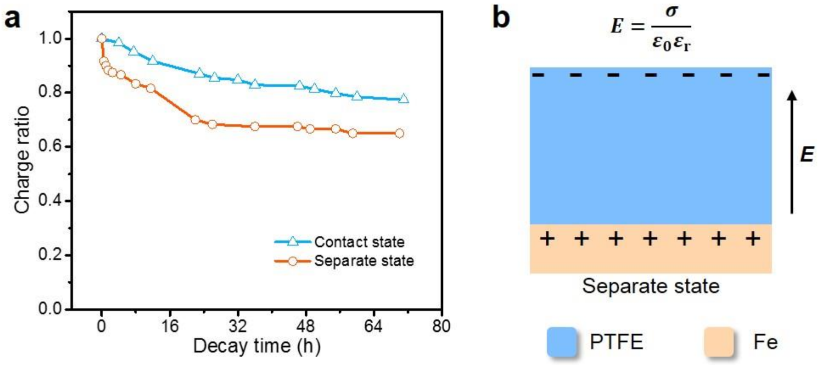

Supplementary Figure 12. Charge decay over time of PTFE film. a Charge decay curve of PTFE film in vacuum condition. **b** Schematic diagram shows the electric field distribution in separate state. Source data are provided as a Source Data file.

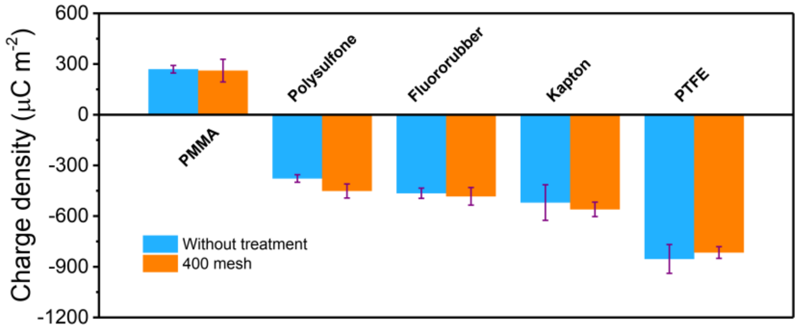

Supplementary Figure 13. TECD of five represented dielectric materials with the counterpart of Fe before and after the Fe electrode was treated by 400 meshes sandpaper. Error bars represent standard deviation, $n = 5$ independent samples. Source data are provided as a Source Data file.

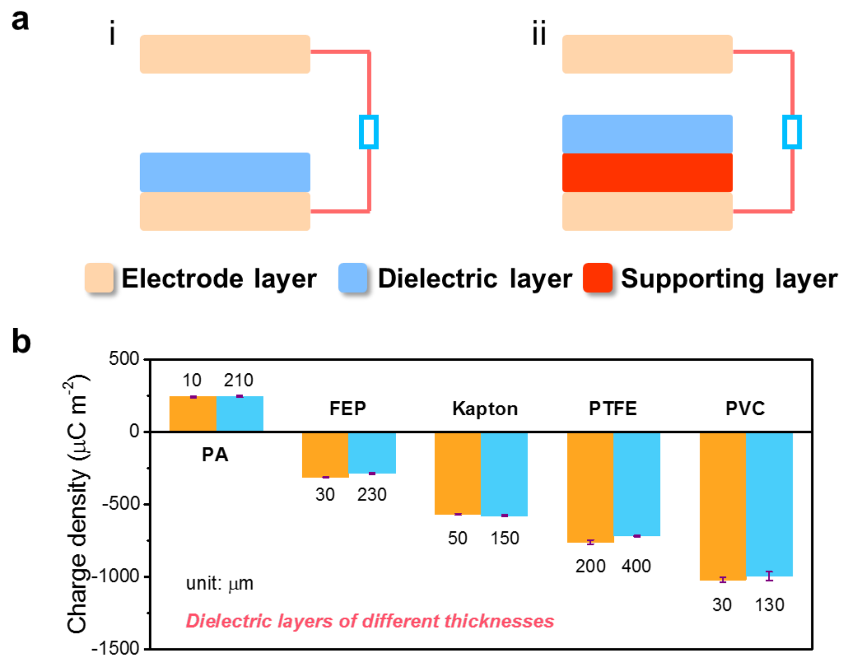

Supplementary Figure 14. TECD of dielectric layers with different thicknesses. a Schematic diagram shows the structure of TENG with different thickness of dielectric layer. **b** The detailed value of TECD of dielectric layers with different thicknesses. Error bars represent standard deviation, $n = 5$ independent replicates. Source data are provided as a Source Data file.

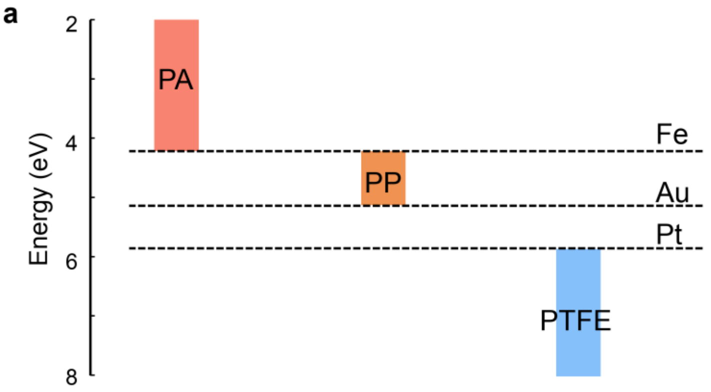

b

Electronegativity increasing (+ → -)

Work function increasing (↓)

	PA	PP	PTFE
Fe	219.0	-110.8	-853.7
Au	270.6	79.5	-347.9
Pt	321.8	98.9	-279.9

Supplementary Figure 15. Work function and effective work function of different materials. a Schematic diagram shows the effective work function of three dielectric materials (PA, PP and PTFE). **b** The detailed value of TECD of nine triboelectric material pairs.

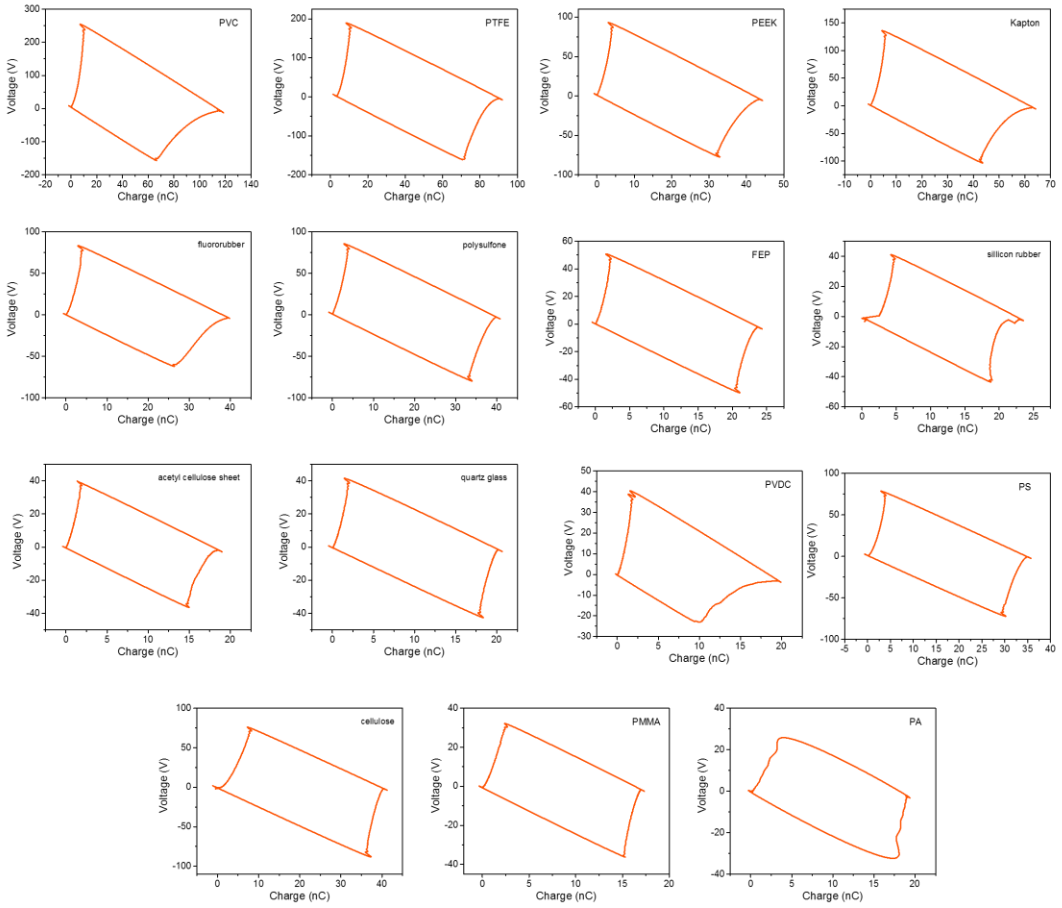

Supplementary Figure 16. Quantified maximized energy density of fifteen represented materials during one working cycle in CS-TENG. Specifically, for cellulose, PMMA and PA, the negative voltage is produced during the separating process. Source data are provided as a Source Data file.

Supplementary Note 1 Summary of the conventional triboelectric series.

When two different materials are brought into contact or rubbered with each other, charges will transfer from one to the other material. Perhaps the obvious and basic question would be asked is which surface will be charged positively or negatively, respectively. Triboelectric series[1], describing the tendency to lose or gain electrons, was considered as the best tool to answer this question, which is an empirical ordering carried out by rubbering one material to against the other. After the first triboelectric series is established by Wilcke in 1757, great efforts have been dedicated to further expand the materials category and the best attempts to determine the polarity of surface charge is still the same method employed by Wilcke. The conventional triboelectric series can only reflect the polarity of triboelectrification instead of the strength of triboelectric performance. In other words, the polarity of transferred charges is preferred rather than the quantity of charges, while the quantity of charges is more important in practical applications.

Despite centuries of research, the scientific understanding of the triboelectric series is still relatively primitive. Evidently, the relatively charging behavior of two materials not only depends on materials' intrinsic properties, but also strongly relates to the environmental factors, such as humidity, gas composition, gas pressure, temperature, breakdown effect, and even the ion or dust adsorption. Namely, the charging phenomenon under practical ambient condition is very complex. Therefore, it is very challenging to scientifically understand the basic mechanism behind triboelectric series based on the results in ambient condition.

Supplementary Note 2 The detailed working mechanism of the CS-TENG.

The CS-TENG is based on the coupling of triboelectrification and electrostatic induction. As the example shown in **Supplementary Figure 1a**, when the metal contacts with the dielectric layer, electrons will transfer from the metal side to the surface of the dielectric layer due to the different electron affinity. If the metal and dielectric layer gradually separate with each other, electrons will flow in external circuit from the back electrode of dielectric layer to the triboelectric electrode, until to the maximum separation distance. Then, if the metal and dielectric layer gradually approach to each other, electrons will flow backward in external circuit, until the metal contacting with the dielectric layer again. On the other hand, there still exists the other case that the dielectric layer is charged positively, as shown in **Supplementary Figure 1b**. When the metal contacts with the dielectric layer, electrons will transfer from the dielectric layer to the metal based on triboelectrification. The dielectric layer will be positively charged. If the metal and dielectric layer gradually separate with each other, electrons will flow in external circuit from the triboelectric electrode to the back electrode of dielectric layer, until to the maximum separation distance. Then, if the metal and dielectric layer gradually approach each other, electrons will flow backward in external circuit, until the metal contacts with the dielectric layer again. This is the whole working mechanism of the CS-TENG.

Supplementary Note 3 Output performance of CS-TENG with and without breakdown effect.

Generally, air breakdown inside the contact surface of TENG is not considered, and the corresponding working mechanism of the CS-TENG is shown in **Supplementary Figure 2a**. When the top electrode contacts with the dielectric layer, electrons will transfer in the contact surface based on triboelectrification. If the top electrode and the dielectric layer are separated far away from each other by the external force, the induced charge density in external circuit (σ_1) will approximately equal to the surface charge density on the dielectric layer (σ_0), forming an induction current of I_1. Previous studies have demonstrated that breakdown effect widely exists in all modes TENGs at atmosphere pressure. In this case, when the top electrode is separated by external force, the high electric field (usually higher than 3 kV mm^{-1}) may cause air breakdown leading to the decrease of surface charge density on the dielectric layer. Therefore, the induced charge density in external circuit will equal to the residual charge density on dielectric surface (σ_2), which will smaller than the charge density by triboelectrification (σ_0). The corresponding output current in external circuit is I_2. It is predicted that the output current of CS-TENG with air breakdown will smaller than the value without air breakdown effect (I_2<I_1).

Supplementary Note 4 Theoretical maximum charge density of CS-TENG.

Paschen's law describes the empirical relationships of breakdown voltage (V_b), gap distance (d) and atmosphere pressure (P), which is shown by

$$V_b = \frac{APd}{\ln(Pd)+B} \quad \text{(S1)}$$

where A and B are constants determined by the composition and the pressure of the gas. For atmosphere at standard atmospheric pressure, A = 273.75 V (Pa m)$^{-1}$, and B = 1.08.

As for a CS-TENG, the gap voltage (V_{gap}) between two contact surfaces of a three-layered TENG in short-circuit can be obtained by theoretical derivation, which is given by

$$V_{gap,SC} = \frac{\sigma t d}{\varepsilon_0(t+d\varepsilon_r)} \quad \text{(S2)}$$

where σ is the triboelectric surface charge density, t the thickness of the dielectric layer, ε_0 (~8.85×10^{-12} F m^{-1}) the vacuum permittivity and ε_r the relative permittivity of dielectric layer (for PTFE, ~2.1). To avoid air breakdown, the V_{gap} must be smaller than V_b at any operation gap distance (d > 0). In other words, the equation should be established,

$$\frac{APd}{\ln(Pd)+B} - \frac{\sigma t d}{\varepsilon_0(t+d\varepsilon_r)} > 0 \quad \text{(S3)}$$

for d > 0. Thus, the maximum surface charge density in short-circuit can be derived as following:

$$\sigma_{max,SC} = \left\{\frac{AP\varepsilon_0(t+d\varepsilon_r)}{t[\ln(Pd)+B]}\right\} min \quad \text{(S4)}$$

From equation (S4) and with the consideration of constant coefficients A and B, the allowed maximum surface charge density at different atmosphere pressures are shown in **Supplementary Figure 6**, where t is 200 μm, 400 μm and 600 μm, respectively, and d is 1 cm.

In near open-circuit condition, V_{gap} is given by

$$V_{gap,OC} = \frac{\sigma d}{\varepsilon_0} \quad \text{(S5)}$$

To avoid air breakdown, the $V_{gap,OC}$ also must be smaller than V_b at any operation gap distance (d > 0). Thus, the maximum surface charge density in near open-circuit can be

derived as following:

$$\sigma_{max,OC} = \left\{\frac{AP\varepsilon_0}{[\ln(Pd)+B]}\right\} min \tag{S6}$$

For a general working distance range of CS-TENG (d: 0~0.01 m), and the corresponding calculated $\sigma_{\mathrm{max,OC}}$ is only 30.7 μC m^{-2} without considering the edge effects of non-ideal parallel capacitor. For a CS-TENG with an area of 10 cm^2, if the edge effects is considered, the calculated $\sigma_{\mathrm{max,OC}}$ is 56.1 μC m^{-2} [2]. In fact, the experimental $\sigma_{\mathrm{max,OC}}$ is only around 40-50 μC m^{-2} (**Fig. 5b**).

Supplementary Note 5 The relationship of induced charges and separating distance.

As shown in **Supplementary Figure 3**, a schematic diagram of the CS-TENG and corresponding physical parameters are presented. When top electrode contacts with the dielectric layer, triboelectric charges will transfer between the contact surface. Generally, the dielectric layer is an insulator, so the triboelectric charges ($-\sigma_0$) will be fixed on the surface. If the periodic external force is applied on the top electrode, electrons will transfer between the two electrodes. Here, the transferred charges in external circuit is Q. The area, thickness and relative permittivity of dielectric layer are S, t and ε_r. For a CS-TENG, the general working distance is within 0.01 m (d: 0-0.01 m). Based on Gauss theorem, the electric field across the dielectric layer can be calculated as follows (without consideration of the edge effect):

$$E_d = -\frac{Q}{S\varepsilon_0\varepsilon_r} \tag{S7}$$

and the electric field across the air gap can be calculated as:

$$E_{air} = \frac{\sigma_0 S - Q}{S\varepsilon_0} \tag{S8}$$

The voltage between the two electrodes can be given:

$$V_{gap} = -\frac{Q}{S\varepsilon_0\varepsilon_r}t + \frac{\sigma_0 S - Q}{S\varepsilon_0}d \tag{S9}$$

In short-circuit condition, the voltage between the two electrodes is zero, so the following equation can be obtained:

$$\sigma = \frac{Q}{S} = \frac{\sigma_0 d\varepsilon_r}{t + d\varepsilon_r} \tag{S10}$$

where σ is the transferred charge density. In our experiments, the represented thickness of dielectric layer is 200 μm, and when ε_r~2, the measured transferred charge density σ in external circuit is 99% of the represented surface charge density σ_0. With the increase of the separation distance x, the transferred charge density in external circuit will gradually close to the surface charge density. If d is 10 cm, the ratio of transferred charge density and surface charge density will up to 99.9%.

Supplementary Note 6 TECD of CS-TENG with different dielectric thickness at various atmosphere pressures.

Previous studies have demonstrated that the CS-TENG with thinner dielectric layer will obtain a high output charge density in atmosphere condition[3,4]. Different from the triboelectrification method, this value was realized by the ion injection method, which is a common method to remove static electricity in many areas and to explore the upper limit of air breakdown in TENG and is also used to improve the performance of TENG. In our work, all the maximum charge densities were realized by triboelectrification, which is helpful for understanding the upper limit of triboelectrification.

Here we calculated the relationship of maximum surface charge density, atmosphere pressure and dielectric thickness as shown in **Supplementary Figure 6a**. We can know that the maximum surface charge density always decreases with the increasing of the dielectric thickness in various atmosphere pressure, which can also be confirmed by experiments as shown in **Fig. 2a** and **Supplementary Figure 7**. With the dielectric thickness increasing, the breakdown effect is more severe and obvious in CS-TENG. Especially in the atmosphere pressure of 300 Pa, the breakdown phenomenon is the most severe condition where the critical breakdown voltage is the lowest value. As shown in **Fig. 2d**, the small peak of output charges in separating process is a solid evidence to demonstrate the existence of air breakdown. **Supplementary Figure 6b** shows the critical gap distance where the air breakdown is about to occur at various atmosphere pressures. It is clearly that the critical gap distance for air breakdown is gradually increasing with the decreasing of the pressure. Thus, it can be expected that when the critical gap distance for air breakdown is larger than the working distance of CS-TENG, and the air breakdown effect can be avoided. We also plotted the relationship of maximum charge density, atmosphere pressure and the dielectric thickness in a three-dimensional diagram (**Supplementary Figure 6c**). The above results indicate that a thinner dielectric layer in CS-TENG often tends to obtain a higher charge density if the breakdown effect exists.

Supplementary Note 7 The special breakdown signal at around 300 Pa.

To interpret the breakdown phenomenon happened in CS-TENG more graphically, based on the data collected during the measurement, a specific charge transfer process and the corresponding output charge curves when air breakdown happened are plotted in **Supplementary Figure 8**. At stage **i** in **Supplementary Figure 8a**, the remaining surface charge density on the dielectric layer is denoted as σ_2 (to keep consistent symbol with the **Supplementary Figure 2**). If the top electrode moves closer to the dielectric layer (stage **ii**), electrons will transfer from the bottom electrode to the top electrode to balance the potential difference between these two electrodes until the top electrode contacting with the dielectric layer (stage **iii**), and the transferred charges (Q_1) can be detected in external circuit. As we know, the remaining surface charge density on dielectric layer is relatively low, so new triboelectric charges will be generated in the contact surface when the top electrode contacts with the dielectric layer (the surface charge density is represented as σ_0). Because the negative and positive charges in contact surface have not been separated yet, there will be no transferred charges in external circuit (stage **iv**). Then, if the top electrode is separated from the dielectric layer, the potential balance in two electrodes will be destroyed, and electrons will transfer from top electrode to the bottom electrode in external circuit (stage **v**). Due to the new triboelectric charges generated in dielectric layer, the induced charges (Q_2) in external circuit will be higher than Q_1. Therefore, the air gap voltage will exceed the critical breakdown voltage again. Generally, the air breakdown theory can be described by Paschen's law as shown in **Fig. 1c**, which also has been demonstrated in CS-TENG in many previous works. The breakdown effect will result in the neutralization of part charges in the top electrode and dielectric layer, so there will be little reversed transferred charges in external circuit (Q_2) to balance the potential in these two electrodes, as shown in stage **vi**. Thus, the special breakdown signal can be detected. Finally, the CS-TENG returns to the initial stage **i**. This is the whole working cycle of CS-TENG with breakdown effect.

Supplementary Note 8 TECD of CS-TENG at different temperatures in 300 Pa.

There are already many research efforts focused on studying temperature effect on output performance of TENG. It is generally considered that high temperature will cause thermionic emission from the dielectric surface, which is negative for the output performance of TENG[5,6]. Given the co-existence of triboelectrification and electrostatic breakdown in atmosphere condition, the output performance of TENG reflects the result of a combination of triboelectrification and breakdown effects. It is difficult to study the temperature effect on triboelectrification. To clearly clarify this point, we chose the atmosphere pressure of 300 Pa and studied the output performance of CS-TENG in various temperatures. Remarkably, the output charges of CS-TENG with different thicknesses all increase with the increasing of temperature as shown in **Fig. 2f**, which is obviously different with the conventional results. Here, in our experiments, the atmosphere pressure of 300 Pa is the critical breakdown point as shown in **Fig. 2a**. According to the breakdown theory, the effects of changing pressure or temperature on breakdown voltage both are reflected as in the relative changes in air density (δ), which is defined as the ratio of the real density to the density under standard atmosphere condition. Given that the atmosphere pressure of 300 Pa is the critical point, any variation of temperature will cause the movement of the breakdown point, and the breakdown voltage will increase (**Supplementary Figure 9**). Therefore, with the increasing of temperature in this condition, the output charges of CS-TENG will increase.

Supplementary Note 9 Thermionic emission effect on charge dissipation.

For a general condition with the existence of air breakdown and the inconspicuous thermionic emission effect (for example, the general working condition of CS-TENG in normal temperature and pressure) (**Supplementary Figure 11a**), the equilibrium surface charge density is a relative low value mainly because of the limitation of air breakdown effect, as shown in **Supplementary Figure 11b(i)**. If the atmosphere pressure decreases to a very low value where the air breakdown effect is avoided (for example, the high vacuum condition) and the thermionic emission effect is inconspicuous, the equilibrium surface charge density will up to a high value as shown in **Supplementary Figure 11b(ii)**. Only at absolute zero degrees, the thermionic emission effect can be avoided, but it takes a long time (generally higher than the period of motion of the TENG) at room temperature. If we raise temperature in high vacuum condition, thermionic emission will cause a part of charge loss, and the equilibrium surface charge density will decrease but still higher than the condition with the existence of air breakdown, as shown in **Supplementary Figure 11b(iii)**.

The surface state model has been proposed to illustrate the thermionic emission effect[5]. In our experiments, the temperature is higher than 0 K, so the distribution of electrons in the metal should be consistent with the statistical distribution of Fermi-Dirac function:

$$f(E) = \frac{1}{e^{[(E-E_F)/k_B T]}+1} \tag{S11}$$

where $f(E)$ is the probability of an electron filling the energy level E, E_F the Fermi level of the metal, k_B the Boltzmann constant, and T the temperature of the metal. With the increase of temperature, the probability that an electron fills the energy level higher than E_F will increase. In our work, the E_F of the metal is higher than the highest occupied surface state of the dielectric E_0 (**Supplementary Figure 11c i**). When the metal contacts with the dielectric, electrons will transit to the surface of the dielectric via the triboelectrification process (**Supplementary Figure 11c ii**). Then, if the metal and the dielectric separated, some of the electrons at higher surface states may transit to the metal and the environment (**Supplementary Figure 11c iii**).

Supplementary Note 10 TECD in vacuum condition with the temperature of around 400 K.

As shown in **Fig. 2i**, the shape of the contact peak seems different from the contact peak in **Fig. 2h**. The possible reason is the enhanced contact intimacy in high temperature. With the increasing of temperature, the dielectric material may become soft and the contact area will also have a slight increase, so the transferred charges have a certain increase with the increase of contact time.

Supplementary Note 11 Charge decay over time of PTFE film

Taking the widely used PTFE film as an example, we tested the decay curves of charge density over time at two conditions in vacuum: (1) contact state and (2) separate state. As shown in **Supplementary Figure 12a**, we can find that the surface charge density of PTFE has a slow decay behavior even in vacuum condition. This is because the absolutely charge density value is very high. But during a periodic contact-motion process at the frequency of a few Hertz, the charge decay behavior can be neglected. We also carefully analyzed the electric field across the dielectric layer in separate state. As we know, in separate state, the negatively charged dielectric layer will induce equal but positive charges in the bottom electrode (**Supplementary Figure 12b**). According to the Gauss's equation ($E = \sigma/\varepsilon_0\varepsilon_r$), when the charge density (σ) is around 100 μC m^{-2} to 1000 μC m^{-2}, the calculated electric field (E) across the dielectric is around 5.4-54 MV m^{-1}, taking the PTFE film as an example (the relative permittivity ε_r is 2.1 and the vacuum permittivity is 8.85×10^{-12} F m^{-1}). The high electric field in separate state would increase the possibility of charge decay from surface to the bulk material.

Supplementary Note 12 The electrode layer

Considering the high hardness and temperature stability of stainless steel 304 (remarked as Fe), it was chosen as the electrode layer for high-temperature experiments. In addition, the conventional used metals such as copper and aluminum may cause materials transfer after friction many times, which might influence the measured surface charge density. In previous studies, we found that Fe not only is very stable but also has good triboelectric performance[7]. In addition, comparing with the original Fe electrode, five represented dielectric materials were used to contact the Fe electrode which is treated by 400 meshes sandpaper, and their corresponding TECD still shows no obvious difference (**Supplementary Figure 13**). These results indicate that the certain surface roughness of Fe electrode in our experiments would not have a big influence on the measured results. To ensure the consistency of the experiments and realize stable output performance, we chose Fe as the electrode layer for all the experiments if not specifically stated.

Supplementary Note 13 How to promise the contact force?

Given that contact force has a close relationship with the output performance of TENG in atmosphere condition[8], it is necessary to analyze the effect of contact force on TECD. In 2017, Wang et al. proposed the limitation factors of surface charge density for TENG (σ_{TENG}), which is vital to understand the output performance of TENG under different conditions, as shown in equation (1) in previous manuscript[9]. Generally, for a TENG working in atmosphere condition, the maximum surface charge density is limited by air breakdown, so the TECD of a CS-TENG will increase gradually with the external force and up to a steady value ($\sigma_{air\ breakdown}$) (the steady value in atmosphere condition in **Fig. 2k**). If the external force increases again, the steady value will not increase. Then, if the atmosphere pressure decreases to a very low value where the air breakdown effect can be avoided, the TECD will increase again with the increasing of external force and up to another steady value. The steady value of surface charge density will be restricted by $\sigma_{triboelectrification}$ or $\sigma_{dielectric\ breakdown}$. Generally, for most of materials, the $\sigma_{dielectric\ breakdown}$ is much higher than the TECD we tested, so the steady value of surface charge density is $\sigma_{triboelectrification}$ (the steady value in vacuum condition in **Fig. 2k**). If the external force increases again, the steady value will not increase. Here, the steady value that don't increase with the increasing of external force is considered as the maximum TECD. Due to the different limitation factors, the tested results in atmosphere and vacuum condition will be different.

Supplementary Note 14 Realization of the triboelectric layer with different thicknesses.

To demonstrate the stability of the test method proposed in this work and study the effect of dielectric thickness on TECD in high vacuum condition, we also did a series of experiments. Five commonly used dielectric materials were chosen for fabricating the CS-TENG. To ensure that the materials' surface of different thicknesses is consistent, we chose the polyimide double-sided adhesive tape as the supporting layer between the triboelectric layer and the electrode layer. As the PVC (thickness: 30 μm) for an example, the schematic diagram is shown in **Supplementary Figure 14a**. There is one layer of polyimide double-sided adhesive tape between the PVC and the bottom electrode layer, so the thickness of the thick "triboelectric layer" is 130 μm. For the Kapton film (thickness: 50 μm), there is still one supporting layer, so the thickness of the thick "triboelectric layer" is 150 μm. Furthermore, we also add two layers of polyimide double-sided adhesive tape as the supporting layer to study the dielectric thickness effect in high vacuum condition. The thickness of the thick "triboelectric layer" of PA (thickness: 10 μm), FEP (thickness: 30 μm) and PTFE (thickness: 200 μm) are 210 μm, 230 μm and 400 μm, respectively. All the results indicate that dielectric thickness has no effect of triboelectric property in high vacuum condition (**Supplementary Figure 14b**), which further demonstrate the feasibility and universality of this method.

Supplementary Note 15 TECD record by triboelectrification

As the invention of TENG in 2012, it has been demonstrated the great potential for application in energy and sensing. Given that the output power is proportional to the square of the surface charge density and the sensor sensitivity is directly proportion to the surface charge density, it is vital to enhance the TECD of TENG and then improve its output performance. In early time, the surface charge density of TENG is about 50 $\mu C\ m^{-2}$. With researchers' efforts, the surface charge density can be improved by materials choice, surface modification (physical and chemical modification), soft and fragmental contact (improve contact intimacy), structural optimization, and so on[10-13]. However, the surface charge density of CS-TENG is restricted by breakdown effect[4,9]. The ultrathin dielectric layer[3] and high atmosphere pressure condition[14] were proposed to suppress air breakdown to some extent, and the surface charge density can be further enhanced. To completely avoid air breakdown effect, high vacuum condition was introduced[9]. The surface charge density can be improved to 660 $\mu C\ m^{-2}$. By further combining with the surface polarization and dielectric polarization, a record-high surface charge density of 1003 $\mu C\ m^{-2}$ is achieved in 2017. The TECD stays at this record, and we still don't know the limit of charge density by triboelectrification.

Supplementary Note 16 Calculation of dielectric breakdown strength

For the CS-TENG, a high electric field will be built across the dielectric layer when the contact electrode is far away from the triboelectric layer. If the electric field caused by TECD is very high, the dielectric breakdown may occur. Given that the charged dielectric layer and the bottom electrode can be regarded as a capacitor (*C*) with opposite charges on their surfaces, the total surface charges can be expressed as

$$Q = C \times V \tag{S12}$$

where *V* is the voltage applied on the dielectric layer, so the electric field (*E*) across the dielectric layer can be described as

$$E = \frac{V}{d} \tag{S13}$$

Therefore, combining with the definition of parallel plate capacitor

$$C = \frac{\varepsilon_0 \varepsilon_r S}{d} \tag{S14}$$

where *S* is the area of the capacitor. Then, the surface charge density can be described as

$$\sigma = \frac{Q}{S} = \varepsilon_0 \varepsilon_r E \tag{S15}$$

The critical value of dielectric breakdown can be calculated as

$$\sigma_{dielectric\ breakdown} = \varepsilon_0 \varepsilon_r E_{dielectric\ breakdown} \tag{S16}$$

For PVC with the thickness of 30 μm, ε_r~4-8 and $E_{\text{dielectric breakdown}}$~50 MV m^{-1} [15,16], so the $\sigma_{\text{dielectric breakdown}}$ can be calculated to be 1770-3540 μC m^{-2}. Here, the TECD of 1250 μC m^{-2} is lower than the critical value for dielectric breakdown, so the TECD represents the $\sigma_{\text{triboelectrification}}$.

Supplementary Note 17 The maximum energy density of CS-TENG in atmosphere and vacuum condition.

As we know, the energy density of TENG (E) is defined by the following equation:

$$E = \int V dQ \tag{S17}$$

where V and Q are the corresponding output voltage and charge density of TENG. For CS-TENG, it is well-known that the maximum surface charge density is limited by the air breakdown effect. Due to the existence of air breakdown effect in atmosphere, the output charges in external circuit will gradually decreases to a steady value with the external load increasing[17], while the output charges keep stable in vacuum condition with the external load increasing. In other words, according to equation (S17), the output energy density in atmosphere condition has a maximum value, while the output energy density in vacuum gradually increases to maximum value and then keeps stable. That's why we draw the schematic diagram showing the maximum energy density in atmosphere and vacuum condition as **Fig. 5c**.

Supplementary Table 1 Triboelectric series of various materials and their triboelectric charge density (TECD)

Number	Materials	Thickness (μm)	Average TECD (μC m^{-2})	Abbr.
1	polyvinyl chloride	30	-1045.48	PVC
2	polytetrafluoroethylene	200	-853.7	PTFE
3	polyether ether ketone	50	-522	PEEK
4	polyimide	50	-519.9	Kapton
5	fluororubber	100	-464.78	
6	polysulfone	250	-377.3	
7	polystyrene	30	-368.12	PS
8	fluorinated ethylene propylene	150	-249.42	FEP
9	sillicon rubber	110	-243.3	
10	polyvinylidene fluoride	110	-242.12	PVDF
11	polyvinyl dichloride	1000	-228.32	PVDC
12	quartz glass	10	-221.3	
13	acetyl cellulose sheet	100	-213.04	
14	polyethylene	50	-196.1	PE
15	acetal	530	-153.2	
16	polyphenylene sulfite	50	-147.42	PPS
17	polydimethylsiloxane	530	-132.36	PDMS
18	acrylonitrile butadiene styrene	120	-122.76	ABS
19	polypropylene	20	-110.76	PP
20	glassfiber	200	-80.94	
21	polyester fabrics	300	-78.88	
22	copy paper	90	-73.96	
23	polyetherimide	520	-52.24	PEI
24	cotton fabrics	210	-41.32	
25	polycarbonate	90	-24.4	PC
26	nitrile rubber	530	-23.52	
27	cotton and linen fabrics	500	-10.36	
28	non-woven fabrics	200	4.6	
29	nylon fabrics	200	7.94	
30	polyformaldehyde	500	11.06	POM
31	thermoplastic polyurethane	10	12.86	TPU
32	polyvinyl alcohol	40	15.24	PVA
33	soda-lime glass	1000	19.76	
34	ethylene-tetra-fluoro-ethylene	100	49.02	ETFE
35	polyethylene terephthalate	100	72.82	PET
36	borosilicate glass	700	125.22	
37	polyamide	10	218.96	PA
38	polymethyl methacrylate	900	269.02	PMM
39	cellulose	530	369.06	
40	polyurethane	900	402.5	PU

References

1. Henniker, J., Triboelectricity in polymers. *Nature* **196**, 474-474 (1962).
2. Zi, Y. L., Wu, C. S., Ding, W. B. & Wang, Z. L. Maximized effective energy output of contact-separationtriggered triboelectric nanogenerators as limited by air breakdown. *Adv. Funct. Mater.* **27**, 1700049 (2017).
3. Zhang, C. L. *et al.* Surface charge density of triboelectric nanogenerators: Theoretical boundary and optimization methodology. *Appl. Mater. Today* **18**, 100496 (2020).
4. Wang, S. H. *et al.* Maximum surface charge density for triboelectric nanogenerators achieved by ionized-air injection: Methodology and theoretical understanding. *Adv. Mater.* **26**, 6720-6728 (2014).
5. Xu, C. *et al.* On the electron-transfer mechanism in the contact-electrification effect. *Adv. Mater.* **30**, 1706790 (2018).
6. Lin, S. Q. *et al.* Electron transfer in nanoscale contact electrification: Effect of temperature in the metal-dielectric case. *Adv. Mater.* **31**, 1808197 (2019).
7. Liu, D. *et al.* Hugely enhanced output power of direct-current triboelectric nanogenerators by using electrostatic breakdown effect. *Adv. Mater. Technol.* **5**, 2000289 (2020).
8. Yang, W. X. *et al.* On the controlled adhesive contact and electrical performance of vertical contact-separation mode triboelectric nanogenerators with micro-grooved surfaces. *Nano Energy* **85**, 106037 (2021).
9. Wang, J. *et al.* Achieving ultrahigh triboelectric charge density for efficient energy harvesting. *Nat. Commun.* **8**, 88 (2017).
10. Wang, J. *et al.* All-plastic-materials based self-charging power system composed of triboelectric nanogenerators and supercapacitors. *Adv. Funct. Mater.* **26**, 1070-1076 (2016).
11. Wang, S. H. *et al.* Molecular surface functionalization to enhance the power output of triboelectric nanogenerators. *J. Mater. Chem. A* **4**, 3728-3734 (2016).
12. Wang, J. *et al.* Sustainably powering wearable electronics solely by biomechanical energy. *Nat. Commun.* **7**, 12744 (2016).
13. Chun, J. S. *et al.* Boosted output performance of triboelectric nanogenerator via electric double layer effect. *Nat. Commun.* **7**, 12985 (2016).
14. Fu, J. J. *et al.* Achieving ultrahigh output energy density of triboelectric nanogenerators in high-pressure gas environment. *Adv. Sci.* **7**, 2001757 (2020).
15. El Sayed, A.M., El-Sayed, S., Morsi, W.M., Mahrous, S. & Hassen, A. Synthesis, characterization, optical, and dielectric properties of polyvinyl chloride/cadmium oxide nanocomposite films. *Polym. Compos.* **35**, 1842-1851 (2014).
16. Thabet, A. & Salem, N. Experimental verification on dielectric breakdown strength using individual and multiple nanoparticles in polyvinyl chloride. *Trans. Electr. Electron. Mater.* **21**, 274-282 (2020).
17. Jiang, C. *et al*. Optimization of triboelectric nanogenerator load characteristics considering the air breakdown effect. *Nano Energy* **53**, 706-715 (2018).
